# The crosstalk among the physical tumor microenvironment and the effects of glucose deprivation on tumors in the past decade

**DOI:** 10.3389/fcell.2023.1275543

**Published:** 2023-11-01

**Authors:** Yingnan Cui, Yuanlin Sun, Dongming Li, Yuzheng Zhang, Yangyu Zhang, Donghui Cao, Xueyuan Cao

**Affiliations:** ^1^ Department of Gastric and Colorectal Surgery, General Surgery Center, The First Hospital of Jilin University, Changchun, China; ^2^ Division of Clinical Epidemiology, The First Hospital of Jilin University, Changchun, China

**Keywords:** glucose deprivation, cancer, tumor microenvironment, cell death, cell survival

## Abstract

The occurrence and progression of tumors are inseparable from glucose metabolism. With the development of tumors, the volume increases gradually and the nutritional supply of tumors cannot be fully guaranteed. The tumor microenvironment changes and glucose deficiency becomes the common stress environment of tumors. Here, we discuss the mutual influences between glucose deprivation and other features of the tumor microenvironment, such as hypoxia, immune escape, low pH, and oxidative stress. In the face of a series of stress responses brought by glucose deficiency, different types of tumors have different coping mechanisms. We summarize the tumor studies on glucose deficiency in the last decade and review the genes and pathways that determine the fate of tumors under harsh conditions. It turns out that most of these genes help tumor cells survive in glucose-deprivation conditions. The development of related inhibitors may bring new opportunities for the treatment of tumors.

## 1 Background

In eukaryotes, metabolic pathways occur within the cytoplasm and mitochondria of cells and most of the energy in animal cells is provided by glucose or fatty acids (Judge and Dodd, 2020). Large amounts of nutrients can provide energy, reductive equivalents, and biosynthetic precursors to support the survival, proliferation, and malignant progression of cancer cells (Brunner and Finley, 2021). However, due to the unlimited proliferation of the cancer cells, the nutrients, such as glucose and glutamine, were always in shortage in the environment, inducing energy stress. And metabolic reprogramming has been widely observed during cancer development under such energy stress, which enables cancer cells to survive and proliferate (Li and Zhang, 2016) and is now recognized as a hallmark of cancer (Hanahan and Weinberg, 2011).

Glucose is the most available nutrient for cancer cells. Cancer cells require a higher glucose supply than normal cells to maintain their rapid proliferation (Yue et al., 2021). Normal tissues use glycolysis to produce about 10% of cellular ATP, of which mitochondria account for 90%. However, more than 50% of the tumor’s cellular energy is produced by glycolysis, with the rest produced by mitochondria. Interestingly, this transition occurs even when the oxygen is enough to support mitochondrial function, which is called Warburg effect (Warburg, 1956). Tumor cells rely on glycolysis for energy production, causing them to consume more glucose, which then accelerates the energy stress (Denko, 2008).

The tumor microenvironment is significant for the proliferation, metastasis of tumor cells. Low glucose, hypoxia, immune escape, low pH, and oxidative stress were the main characteristics of the tumor microenvironment. In this paper, we reviewed the crosstalk among the above characteristics and the effects of glucose deprivation on tumorigenesis and development in the past decade.

## 2 The main characteristics of the physical tumor microenvironment (TME)

TME is complicated, including a social microenvironment and a physical microenvironment. The social microenvironment includes all non-cancerous host cells in the tumor, including fibroblasts, adipocytes, endothelial cells, neurons, adaptive and innate immune cells, as well as its non-cellular components, including extracellular matrix (ECM), and soluble products, such as chemokines, cytokines, growth factors, and extracellular vesicles. All organisms living in the social microenvironment will encounter hypoxia, immune surveillance, low pH, oxidative stress, nutrient deprivation and competition, and physical pressure which together make up the physical microenvironment (Sun et al., 2018; Xiao and Yu, 2021).

**FIGURE 1:**
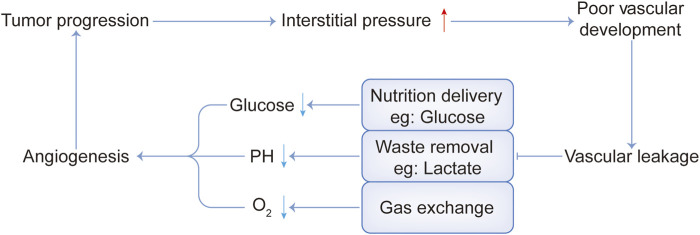
Interaction between several harsh physical tumor microenvironment conditions.

As we know, cancer-associated fibroblasts (CAFs) are considered to be key mediators of interactions between malignant tumor cells and their microenvironment (Barron and Rowley, 2012; Franco and Hayward, 2012). Quiescent or resting fibroblasts are inert, spindle-shaped single cells embedded in the interstitial space of the ECM. When the quiescent fibroblasts are activated, they gain further secretory phenotypes, such as generating cytokines and chemokines, recruiting immune cells, synthesizing ECM, and exerting physical forces to modify tissue structure (Kalluri, 2016). Then ECM is remodeled and the interstitial pressure rises, impeding the activity of the vascular system (Jain et al., 2014; DuFort et al., 2016). In addition, tumors have dilated and tortuous blood vessels with uneven vascular density and diameter (Katayama et al., 2019). Therefore, poor vascular development and vascular leakage lead to the failure of nutrient delivery, metabolic waste removal difficulties, and obstruction of gas exchange. This causes nutrient deficiency such as glucose, metabolic waste deposits such as lactate, and a state of hypoxia, as shown in Figure 1.

### 2.1 Hypoxia

In primary tumors, hypoxia may occur within the mass due to impaired vascularization. Hypoxia is common in locally advanced solid tumors and has become an important factor in tumor physiology due to its ability to promote tumor initiation, progression, and treatment resistance (Carnero and Lleonart, 2016). Hypoxia will further increase the dependence of tumor cells on glycolysis, upregulating glucose transporters such as GLUT1 and enzymes associated with glycolytic pathways (Buono and Longo, 2018; Al Tameemi et al., 2019). Hypoxia also increases the levels of hypoxia-inducible transcription factors 1α (HIF-1α) and HIF-2α, thereby upregulating glycolysis (Shaw, 2006; Semenza, 2010; Kierans and Taylor, 2021). As a result, more glucose is consumed, which can lead to glucose deficiency. In addition, the expression of HIF-mediated gene products promotes vascular network regeneration to reverse hypoxia. However, the newly formed blood vessels are often irregular and distorted, which are inefficient in material transport and eventually lead to nutrient limitation (Brahimi-Horn et al., 2007; Rey and Semenza, 2010), including glucose limitation.

### 2.2 Immune escape

Immune cells in TME include early immune infiltrating cells such as lymphocytes, natural killer cells (NK), macrophages, and dendritic cells (DCs). These cells are suppressed by the action of immunosuppressive cells, such as regulatory T cells (Tregs), myeloid-derived suppressor cells (MDSCs), and type 2-polarized macrophages (M2) (Pitt et al., 2016). Nutritional competition and metabolic interactions between cancer cells and T cells are thought to be key drivers of tumorigenesis. Fast-growing cancer cells will consume most of the nutrients, and immune cells must metabolically adapt to these changes to perform necessary functions when subjected to local nutrient deprivation (Chang et al., 2015). On the other hand, metabolic changes occurring in cancer cells will affect the function of immune cells and promote immune evasion of tumors (Cassim and Pouyssegur, 2019). Glucose-deficient tumor microenvironment limited aerobic glycolysis, and altered the production of IFN-γ, thereby impairing proliferation, cytokine production, and cytolysis of tumor-infiltrating T cells (Cham and Gajewski, 2005; Cham et al., 2008; Chang et al., 2015; Ho et al., 2015).

AMP-activated protein kinase (AMPK) is an indirect glucose sensor and mTOR complex 1 (mTORC1) is an important metabolic regulator controlling NK cell differentiation, shaping T-cell differentiation, and regulating the function of antigen-presenting DCs. The altered AMPK-mTORC1 signaling pathway due to glucose limitation would suppress NK cell and inflammatory T cell responses, promoted Treg differentiation, and increased DC pro-inflammatory output (Kedia-Mehta and Finlay, 2019). Taken together, glucose is of great importance to immune surveillance.

### 2.3 Acidic environment

Acidosis, a constant stressor of most tumor cells, is formed by the fermentation of glucose into lactate in normoxic or hypoxic regions (Ordway et al., 2021). Cancer cells undergo a high rate of glycolysis despite aerobic conditions, leading to glucose consumption and increased lactate production in tumor cells (Gwangwa et al., 2018). To maintain the homeostasis of intracellular pH levels, cancer cells need to actively transport lactate into the extracellular space (Yan et al., 2019). The major players in cancer extracellular acidification are the monocarboxylate transport (MCT) proteins, specifically MCT1/4, whose expressions are elevated to move the accumulation of lactic acid and H^+^(Ordway et al., 2021).

H^+^ ions flow from the tumor into adjacent normal tissue along a concentration gradient, causing tissue remodeling. The resulting acidic environment is also toxic to normal cells and promotes the proteinase degradation of ECM. However, cancer cells invaded and occupied the degrading matrix of normal cells (Gottfried et al., 2012; Estrella et al., 2013), increased angiogenesis through the release of VEGF, and suppressed the immune response to tumor antigens. It has been suggested that an acidic pH is essential for tumorigenesis, invasion, and metastasis (Gwangwa et al., 2018).

### 2.4 Oxidative stress

The glucose deprivation and hypoxia reduced ATP production and accelerated the production and accumulation of reactive oxygen species (ROS) (Ren and Shen, 2019). Glucose deprivation can also induce oxidative stress and mitochondrial dysfunction in rat pheochromocytoma (PC12) cells and human cancer cells, with cytotoxic effects due to ATP depletion and ROS accumulation (Liu et al., 2003; Ahmad et al., 2005). It has been suggested that oxidative stress induced by glucose deprivation activates gene expression and signal transduction in tumors (Blackburn et al., 1999). Glucose depletion can regulate multiple cellular processes by activating miRNA expression through oxidative stress and inhibition of histone deacetylation (Druz et al., 2012).

Under chronic metabolic oxidative stress conditions, cancer cells may upregulate glucose metabolism and produce more NADPH and pyruvate to prevent their toxicity (Spitz et al., 2000; Simons et al., 2009). Nicotinamide phosphoribosyltransferase (NAMPT), a rate-limiting enzyme involved in NAD^+^ biosynthesis, protects tumor cells from glucose deprivation-induced oxidative stress by maintaining NADPH levels (Hong et al., 2016). It has been suggested that oxidative stress induced by glucose deprivation is associated with aggresome formation and autophagy in cultured cardiomyocytes (Marambio et al., 2010). In addition, oxidative stress induced by glucose starvation triggers the LKB1-AMPK signaling pathway to facilitate selective autophagy, thereby enhancing Keap1 degradation and the Nrf2 activation (Endo et al., 2018). Hexokinase-II (HK-II) regulates glucose starvation-induced autophagy by binding to and inhibiting the autophagy suppressor, mTORC1, switching cells from an energy-sufficient metabolic economy to a conserved economy under starvation (Roberts et al., 2014).

### 2.5 Crosstalk among the physical tumor microenvironment

**FIGURE 2:**
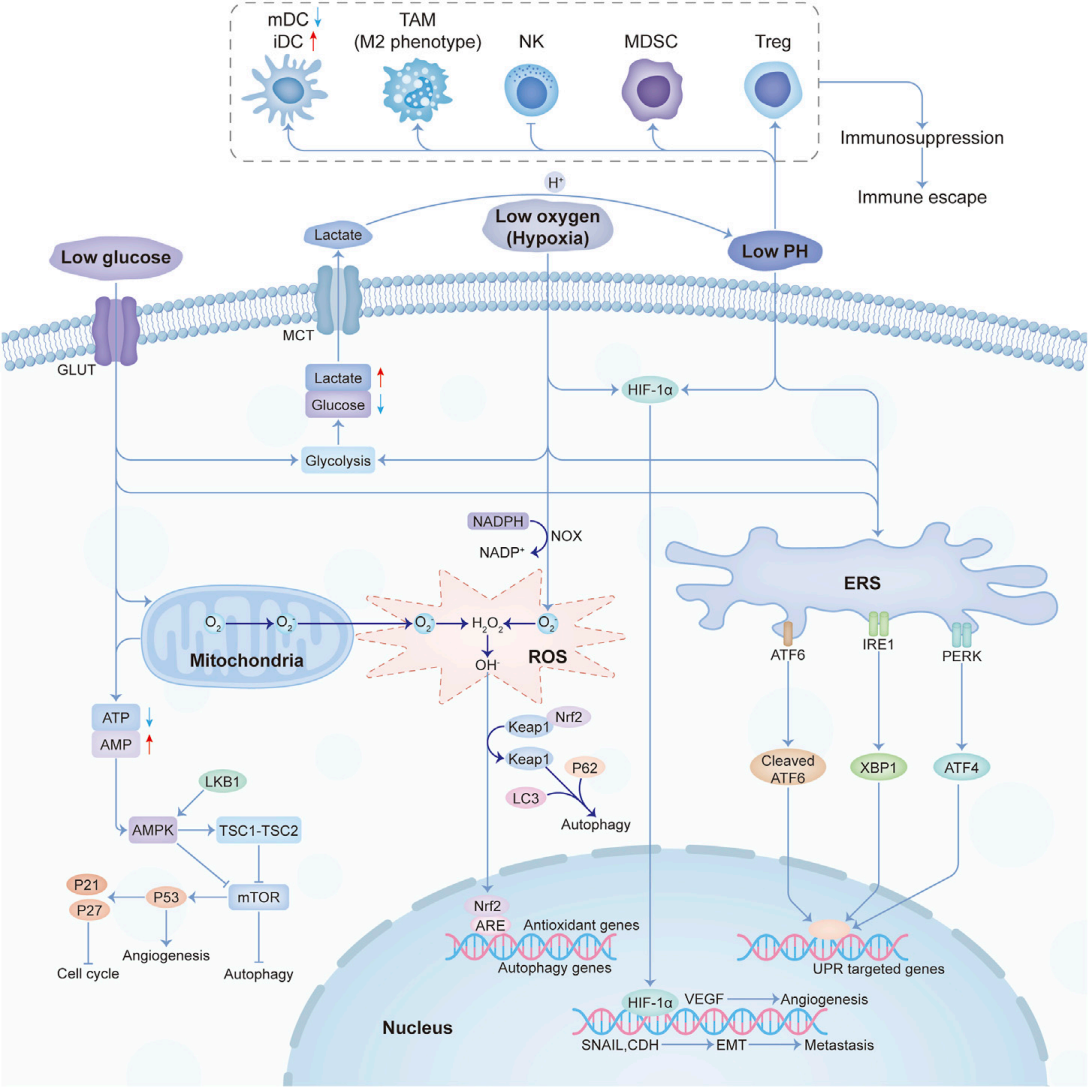
The crosstalk among the main constituents of the physical tumor microenvironment. Abbreviations: mDC, mature dendritic cell; iDC, immature dendritic cell; TAM, tumor-associated macrophages; NK, natural killer; MDSC, myeloid-derived suppressor cell; Treg, T regulatory.

Low glucose, hypoxia, immune escape, low pH, and oxidative stress were the main characteristics of the physical tumor microenvironment, and they influence each other mutually. Low glucose will promote the proliferation of vascular endothelial cells by inhibiting VEGFR2 O-GlcNAcylation and its proteasomal degradation (Deng, 2023). Hypoxia not only promotes angiogenesis through HIF-1-dependent processes (Augustin et al., 2020) but also through lactic acidosis (Singh et al., 2023). At the same time, the hypoxic response will also cause the enhancement of tumor glycolysis and lactic acid deposition. The buildup of lactic acid acidifies the TME and then affects the recognition and response of the immune system to the tumor. Under the condition of nutrition restriction, tumor immunity will also be influenced (Lyssiotis and Kimmelman, 2017). And the crosstalk among different constituents in the physical microenvironment was shown in Figure 2.

### 2.6 Physical tumor microenvironment derived important biological processes

#### 2.6.1 Endoplasmic reticulum stress (ERS)

The protein-folding capacity of the endoplasmic reticulum (ER) in tumor cells and infiltrating immune cells are altered under harsh microenvironmental conditions, which promotes the accumulation of misfolded or unfolded proteins, leading to ERS (Chen and Cubillos-Ruiz, 2021). Hypoxia will trigger ERS by affecting disulfide bond formation and protein folding to different degrees (May et al., 2005; Koumenis and Wouters, 2006). Glucose restriction interrupts the hexosamine biosynthetic pathway (HBP), which affects protein glycosylation and protein folding (Ricciardiello et al., 2020; Lam et al., 2021). Intracellular ROS accumulation (Shimizu and Hendershot, 2009) and acidosis (Maeyashiki et al., 2020) also readily inhibit the protein-folding capacity of the ER, thus triggering a sustained ERS response. Then intracellular signal transduction pathways are activated, which is called the unfolded protein response (UPR) (Ron and Walter, 2007).

Three different types of ER stress transducers were identified to be involved in this process, including activating transcription factor 6 (ATF6), inositol requiring protein 1 (IRE1), and protein kinase RNA (PKR)-like ER kinase (PERK). To alleviate the accumulation of misfolded proteins, signal transduction events are induced by these transducers. A process called ERAD (ER-associated degradation), which stimulates the retrograde transport of misfolded proteins from the ER into the cytosol for ubiquitination and destruction, is involved (Sano and Reed, 2013). In addition, four protein kinases, including PKR, PERK, general control nonderepressible 2 (GCN2), and heme-regulated inhibitor (HRI), can phosphorylate eIF2α in response to stressors (Nakagawa and Ohta, 2019). PKR, GCN2, and HRI can be independent of the ERS pathway, and this part of the UPR is called the integrated stress response (ISR) (Ron and Walter, 2007). In addition to coordinating ER function to restore homeostasis, this series of reactions also alter immune cell function in TME to influence tumor malignant progression. ER-stressed tumor cells alter NK cell-mediated tumor recognition, release other factors to recruit or alter myeloid cell function, as well as regulating T cell-mediated tumor growth, metastasis, and response to immunotherapy. Moreover, activation of UPR may help promote a dormant state for stressed tumor cells and maintain their initial survival (Chen and Cubillos-Ruiz, 2021).

#### 2.6.2 Autophagy

Autophagy plays a dynamic inhibitory or promoting role in different stages of tumor development. As a survival pathway and quality control mechanism, autophagy can prevent tumorigenesis and inhibit cancer progression in early tumorigenesis. Once a tumor progresses to an advanced stage and is subjected to environmental stress, autophagy will act as a dynamic degradation and recycling system that contributes to the survival and growth of established tumors while enhancing cancer aggressiveness by promoting metastasis (Li et al., 2020a). Under metabolic stress conditions such as glucose deficiency, tumor cells in TME promote cell survival by activating autophagy, which is closely related to the mTOR and AMPK pathways (Kimmelman and White, 2017). Hypoxia can activate AMPK, HIF-1α, or ATF4, and induce downstream gene expression to activate autophagy to mediate cell survival (Rouschop et al., 2010; Hu et al., 2012; Di Conza et al., 2017a). Oxidative stress can induce autophagy through nuclear factor-κb (NF-κB) or LKB1-AMPK, and activated autophagy can also promote antioxidant response through Keap1-Nrf2 to alleviate oxidative stress (Alexander et al., 2010; Taguchi et al., 2012; Song et al., 2017). Moreover, autophagy is involved in the survival, apoptosis, and differentiation of immune cell subsets (Xia et al., 2021). Targeting the autophagy pathway to improve the efficacy of immunotherapy will be a promising area.

## 3 The effects of glucose deprivation on tumorigenesis and development

**FIGURE 3:**
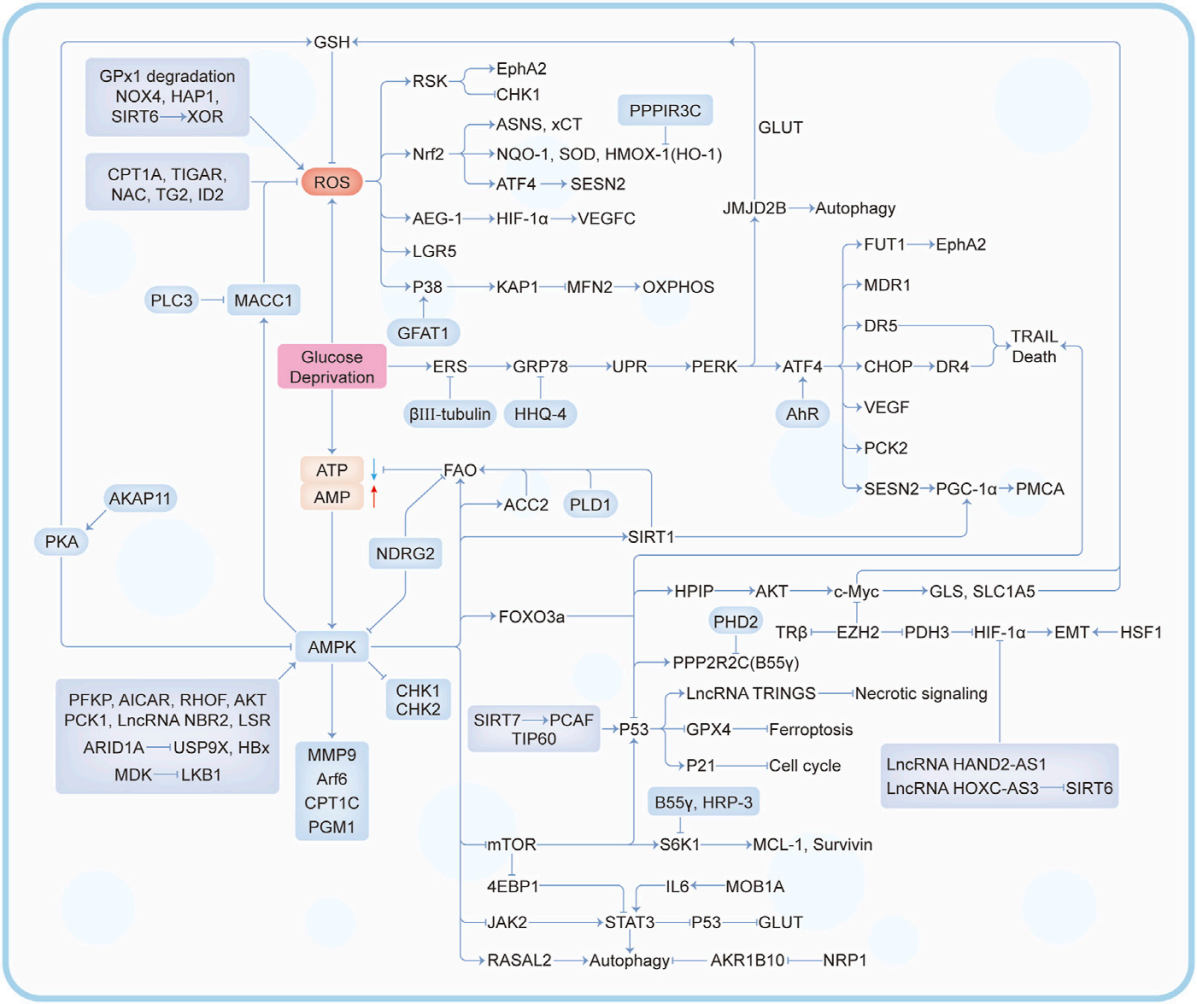
A series of regulatory mechanisms and related molecules triggered by glucose deprivation.

**FIGURE 4:**
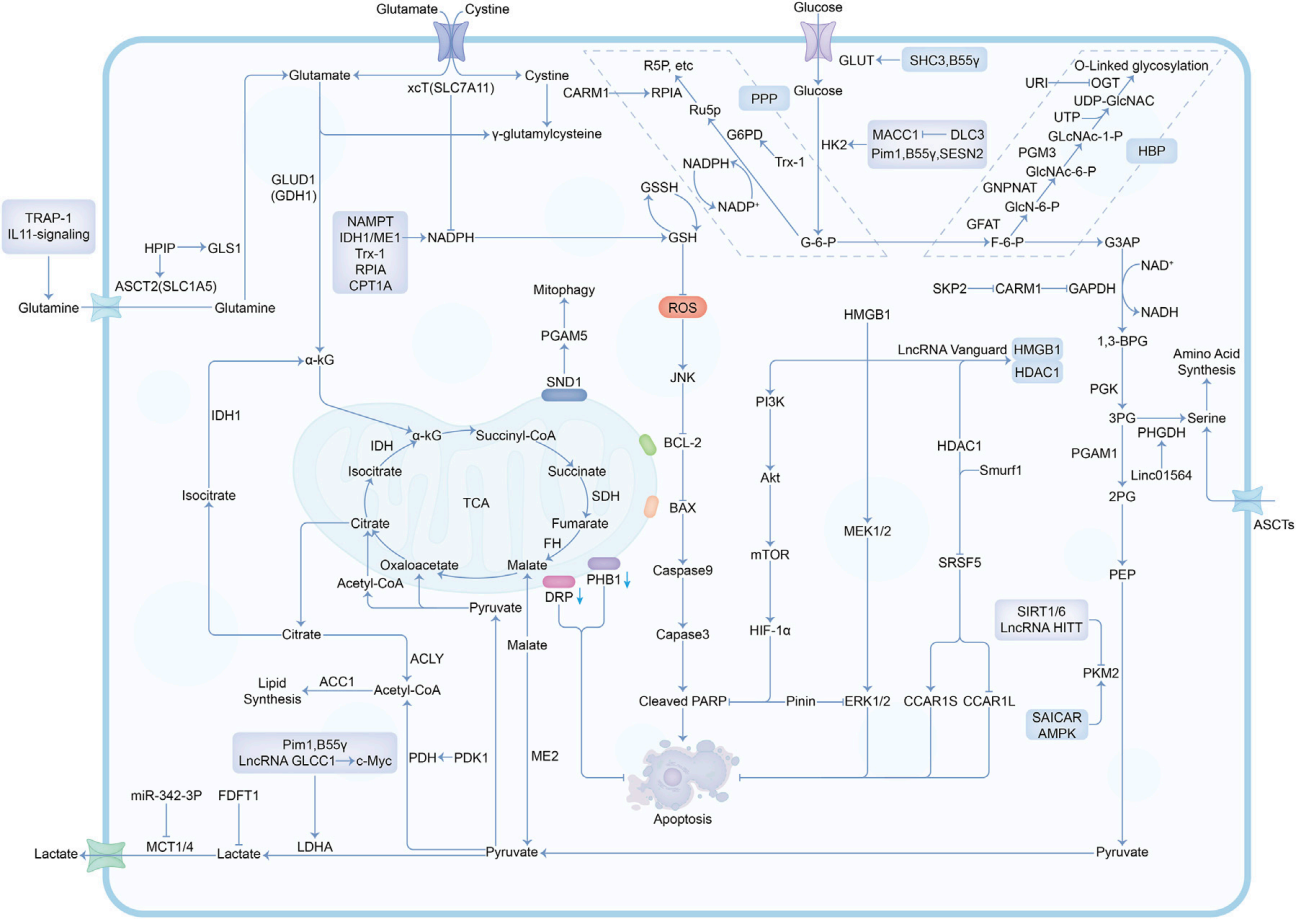
The mechanisms and molecules related to metabolic pathways under glucose deprivation conditions. Abbreviations: G-6-P, Glucose-6-phosphate; F-6-P, Fructose-6-phosphate; G3AP, Glyceraldehyde-3-phosphate; 1,3-BPG, 1,3-Biphosphoglycerate; 3PG, 3-Phosphoglycerate; 2PG, 2-Phosphoglycerate; PEP, Phosphoenolpyruvate; Ru5P, Ribulose-5-phosphate; R5P, Ribose-5-phosphate; GlcN-6-P, Glucosamine-6-phosphate; GlcNAc-6-P, N-acetylglucosamine-6-phosphate; GlcNAc-1-P, N-acetylglucosamine-1-phosphate; PPP, Pentose Phosphate Pathway; HBP, Hexosamine Biosynthesis Pathway.

In the past decade, there have been a lot of cancer studies on glucose deprivation. Among these studies, researchers have found many important molecules for tumor survival or death, which play a direct or indirect role in response to energy stress, as shown in Figures 3, 4. Several tumor types with a large number of studies are shown in Supplementary Table S1.

### 3.1 Pan-cancer

Studies involving a variety of tumors have suggested that many molecules participated in the response to energy stress. Glucose deficiency necessarily affects metabolic homeostasis and the enzymes involved in glycolysis. SRSF5 (Serine and arginine-rich splicing factor 5), a member of the serine/arginine (SR)-rich family of pre-mRNA splicing factors, promotes the production of CCAR1S proteins by alternative splicing CCAR1 which enhances glucose consumption and acetyl-coA production, thus promoting tumor growth (Chen et al., 2018). SAICAR can alter cellular energy levels, glucose uptake, and lactate production. SAICAR-PKM2 interaction promoted cancer cell survival under glucose limitation conditions (Keller et al., 2012). In addition, many molecules related to AMPK as well as the production of GSH, NADPH, and ATP are associated with glucose deprivation. For example, MDK inhibits both basal and stress-induced activation of AMPK by disrupting the LKB1-STRAD-Mo25 complex (Xia et al., 2022). AMPK will also phosphorylate the CHK1 domain for subsequent ubiquitination and degradation, which causes DNA mutagenesis and affects the cell cycle and tumor progression (Ma et al., 2019). ME1 produces NADPH in the cytoplasm, which acts as a reducing agent and affects macromolecular biosynthesis and redox homeostasis (Murai et al., 2017). PLD1 inhibition will block fatty acid oxidation (FAO) and inhibits ATP production, increasing ROS and leading to cancer cell death (Cai et al., 2016). PKA can regulate different genes involved in glutaminolysis by coordinating their transcription (Palorini et al., 2016). Such a coordinated regulation applies to oncogenes such as c-myc (Sun et al., 2015). Besides, some molecules are also involved in cell death-related pathways, including TRAIL receptor-related apoptosis (Iurlaro et al., 2017), necrosis (Khan et al., 2017), and disulfidptosis (Liu et al., 2023).

### 3.2 Liver cancer and pancreatic cancer

In liver cancer, many molecules respond to energy stress by regulating proteins related to glycolysis and glutaminolysis. SESN2 affects glycolysis by destabilizing HK2 mRNA (Kumar et al., 2018; Li et al., 2023a). GDH1 can drive glutamine-derived carbon into the TCA cycle in response to glucose starvation (Zhou et al., 2022). RHOF promotes the Warburg effect by upregulating the expression and function of several glycolytic enzymes, including GLUT1, HK2, PDK1, and LDH (Li et al., 2021a). Meanwhile, there are also some regulatory pathways related to AMPK, such as autophagy and FAO (fatty acid oxidation), which not only affect cell fate but also cause changes in ATP production. SKP2 promotes HCC (hepatocellular carcinoma) progression and its autophagy-induced nuclear function via CARM1 and AMPK(Wei et al., 2018). GPx1 induces protective autophagy in PDA (pancreatic ductal adenocarcinoma) cells under extreme glucose starvation (Meng et al., 2018). HBx promotes FAO in HCC cells in the absence of glucose, thereby maintaining NADPH and ATP levels (Wang et al., 2016a). Upregulation of CPT-1A increases intracellular ATP required for PDA cell survival (Luo et al., 2016). Besides, some stress responses are involved in EMT (epithelial-mesenchymal transition) and angiogenesis, which are undoubtedly another interpretation of metastasis to facilitate tumor escape from harsh energy stress conditions. HSF1 is required for EMT-related migration of HCC cells under low glucose conditions (Liu et al., 2016). In human hepatoblastoma HepG2 cells, the AhR pathway will induce VEGF expression (Terashima et al., 2013). In addition, the researchers examined non-polar and lipid metabolites in the microenvironment. By characterizing polar small molecule nutrients in PDAC and LUAD tumors, we can identify the metabolic liabilities of cancer cells, which ultimately translates into more effective treatments (Sullivan et al., 2019). For example, one study suggests that forced hyperglycemia may provide a new treatment strategy for pancreatic cancer sensitization to chemotherapy (Vaziri-Gohar et al., 2023). Cell metabolism can respond to and adapt to environmental nutrient levels. In different nutritional environments, cancer cells can change their metabolic needs and response to drugs (Schug et al., 2015; Muir et al., 2017; Vaziri-Gohar et al., 2018).

### 3.3 Breast cancer and lung cancer

When faced with energy stress, the metabolism of the tumor is altered. Breast cancer cells induce the expression of proto-oncogene HPIP through the AMPK-FOXO3a pathway. HPIP reconnects glutaminolysis by controlling the expression of solute carrier family 1 member 5 (SLC1A5) and glutaminase (GLS) genes (Penugurti et al., 2021). KAP1 Ser473 phosphorylation in glucose-starved breast cancer cells restricts mitochondrial hyperfusion, leading to reduced oxidative phosphorylation (OXPHOS) and ROS production (Cheng et al., 2016). In addition to mechanisms related to metabolism, the stress mechanisms also include ERS and oxidative stress. Glucose-regulated protein 78 (GRP78) responds to ERS by inducing the unfolded protein response (UPR) to support cellular homeostasis and survival under stress conditions (Xiao et al., 2019). PPARδ regulates breast cancer cell survival under harsh microenvironmental conditions by reducing oxidative stress (Wang et al., 2016b). Nrf2 is a major regulator of antioxidant responses, and its increased activity protects breast cancer cells during glucose deprivation (Walker et al., 2018). Meanwhile, a vicious cycle between AMPK inactivation and ROS exists in LKB1-mutant non-small cell lung cancer (NSCLC) cells, which are susceptible to glucose starvation leading to cell death (Ren et al., 2021). Similarly, we noticed that many molecules were strongly associated with various types of cell death in these studies. Entosis is a cannibalistic process between cells that can resist metabolic stress, PCK2 affects the entosis process by controlling protein glycosylation (Hyroššová et al., 2022). Glucose deprivation evokes ERS and induces ORP150 expression to inhibit apoptosis and senescence of breast cancer cells (Krętowski et al., 2013). Glucose deprivation can also trigger ZBP1-dependent necroptosis in breast cancer (Baik et al., 2021). Apart from PHB1 (Raut et al., 2019) and NDRG2 (Kim et al., 2014) are associated with apoptosis under energy stress conditions in breast cancer, 4EBP1 can sense extracellular glucose deprivation and initiates lung cancer cell death (Wang et al., 2022).

### 3.4 Gastric cancer and colorectal cancer

In gastrointestinal cancer, many molecules affect cell fate under energy stress by regulating metabolism. The molecules, including lncRNA GLCC1(Tang et al., 2019), Pim1 (Zhang et al., 2018), and JMJD2B (Fu et al., 2018), are upregulated under glucose starvation in colorectal cancer (CRC) cells to support cell survival and proliferation by enhancing glycolysis. In gastric cancer, glucose starvation inhibits the malignant behavior through the miR-216a-5p/FDFT1 axis (Zhao et al., 2021). Meanwhile, gastric cancer cells can escape metabolic stress through DLC3/MACC1 axis (Lin et al., 2019). In this process, DLC3 expression is decreased and MACC1 expression is increased. MACC1 then promotes the Warburg effect by upregulating a series of glycolytic enzymes (Lin et al., 2015). Tumor cells can also regulate migration and invasion to escape from energy stress. HMGB1 is involved in the stimulation of colonic myofibroblast migration and invasion under glucose deprivation (Sharma et al., 2016). Acss2/HIF-2 signaling is activated by glucose deprivation in colon cancer cells and is essential for cell migration and invasion (Garcia et al., 2023). In addition, some molecules are also associated with apoptosis. For example, HAP1 not only inhibits gastric cancer cell migration and invasion but also promotes cell death during glucose deprivation (Qu et al., 2023). In contrast, HIF-1α signaling was activated to acquire anti-apoptotic properties in colon cancer (Nishimoto et al., 2014). Another way to promote cell survival under energy stress is to enhance NADPH production, through which Trx-1 (Lu et al., 2022) and RPIA (Guo et al., 2020) support ROS clearance.

### 3.5 Glioma and other types of cancer

When glioma undergoes glucose deprivation, in addition to maintaining metabolic homeostasis by regulating glycolysis (Azzalin et al., 2020) and glutaminolysis (Stuart et al., 2023), many molecules mediate oxidative stress and apoptosis pathways to affect the outcome. ID2 inhibits ROS production, reduces mitochondrial damage, and enhances tumor cell survival (Zhang et al., 2017b). Meanwhile, overexpression of xCT (Yamaguchi et al., 2020; Yamamoto et al., 2021) or SIRT6(Sheikh et al., 2018) can induce ROS accumulation and cell death. In addition, there are many pathways involved in other types of cancer. MAT2A can promote the growth of cervical cancer cells under glucose deprivation by mediating the methylation of programmed cell death protein 6 (PDCD6) (Luo et al., 2022). LSR promotes ovarian cancer cell survival and tumor growth through the LKB1-AMPK pathway (Takahashi et al., 2021). GLUT1 protects prostate cancer cells from oxidative stress induced by glucose deprivation (Gonzalez-Menendez et al., 2018). GPX4-dependent ferroptosis was significantly enhanced by AMPK activation in renal cancer cells upon glucose deprivation. Moreover, in the presence of glucose deficiency, ASNS (Fang et al., 2020), and stathmin1 (Wang et al., 2021) promote the migration and invasion of esophageal squamous cell carcinoma (ESCC) cells and gallbladder carcinoma (GBC) cells, respectively.

## 4 Promising direction: Drug research

The tumor microenvironment is very complex and contains a variety of cells and components. Different cell subpopulations in the tumor microenvironment have different functions and influence tumor development through multiple mechanisms. Therapies that directly act on tumor cells have many shortcomings, such as tumor heterogeneity, genetic instability of cancer cells, drug resistance of cancer cells, etc. Targeting other components of the tumor microenvironment has gradually become the focus of research. This includes all non-cancerous host cells in the tumor, such as endothelial cells, fibroblasts, fat cells, immune cells, as well as its non-cellular components, including the extracellular matrix (ECM) and soluble products, such as cytokines, chemokines, growth factors, etc. Immune cells typically include T and B lymphocytes, tumor-associated macrophages (TAM), dendritic cells (DC), natural killer (NK) cells, neutrophils, and myeloid suppressor cells (MDSC).

There are many kinds of drugs. For example, for CAF and ECM, there are FAP monoclonal antibody, vitamin D analogue, PDGFR inhibitor, CXCR4 receptor antagonist and so on. Drugs that target the monocyte/macrophage populations are CCR2 blocking antibodies or antagonists, PI3K-γ inhibitor, CXCR1/2 antagonist, CD40 agonist antibodies, etc. Drugs targeting tumor vasculature include VEGF/VEGFR inhibitors, ANG inhibitors and so on. Despite the multitude of targets, many clinical trials targeting TME have failed to show promising efficacy in cancer patients. The only exception is immunotherapy, including immune checkpoint blocking therapy, such as anti-PD1/PDL1 therapy, have shown significantly higher efficacy in treating tumors with pre-existing anti-tumor immunity. Moreover, people are beginning to realize that combination therapy may have greater value. With the development of cutting-edge technologies such as single-cell multi-omics and artificial intelligence, we will decipher TME through multi-omics profiling to further improve our understanding of TME. (Bejarano et al., 2021; Xiao and Yu, 2021).

Glucose deprivation, one of the characteristics of the tumor physical microenvironment that we focused on, we learned there are some preclinical and clinical studies in which combinations of drugs are tested in which the effect due to glucose deprivation is exploited. Under nutritional restrictions, allosteric inhibitors of wild-type isocitrate dehydrogenase 1 (wtIDH1) can be lethal to cancer cells (Vaziri-Gohar et al., 2022). Buformin, a UPR (unfolded protein response) inhibitor, effectively reduces the survival of kidney cancer cells with sensitive and resistant phenotypes under glucose deprivation conditions (Isono et al., 2014). Metformin, a drug used in the treatment of type II diabetes, works synergistically with glucose deprivation to inhibit triple-negative breast cancer cell proliferation by activating pro-apoptotic molecules through UPR (Li et al., 2023c). One study suggested that the use of insulin to treat hyperglycemia improved skeletal muscle protein and amino acid metabolism in cancer patients after major surgery (Biolo et al., 2008). Moreover, it has been suggested that a drug combination of niclosamide and quinacrine can inhibit melanoma proliferation and glucose intake (Li et al., 2023c). SCIC2.1, an activator of SIRT1, can promote energy homeostasis and alleviate metabolic stress in liver cancer cells subjected to glucose deprivation (Varghese et al., 2023). GBS-01, an extract from the fruit of Arctium lappa L, has been reported to attenuate the tolerance of cancer cells to glucose deprivation and exert anti-tumor activity. It plays a role in patients with gemcitabine refractory advanced pancreatic cancer (Ikeda et al., 2016). LKB1 is a key sensor of metabolic stress, including hypoxia and glucose deprivation, which are common in the tumor microenvironment exacerbated by antiangiogenic therapy. LKB1 has the potential to predict the sensitivity of advanced non-small cell lung cancer to bevacizumab (Bonanno et al., 2017). In addition, in liver cancer, another option other than surgery to kill the tumor is to block the blood supply through a process called embolization. This process deprives tumor cells of oxygen and nutrients such as glucose (Chao et al., 2016). This is actually related to the effects of glucose deprivation. anti-angiogenic therapy can also cause harsh hypoxia and glucose limiting microenvironments (Boso et al., 2023), and GLUT inhibitors can mimic glucose starvation (Yang et al., 2023).

## 5 Conclusion

The tumor microenvironment is closely related to the external conditions faced by tumors, and it has a complex and huge regulatory network. Starting with glucose deprivation, we can see the correlation between several features of the tumor microenvironment. At the same time, we have also learned many genes and pathways that determine the fate of tumor cells under glucose deprivation conditions. In addition to cell death due to stress, there are many mechanisms mediating tumor survival. Targeting these molecules and designing corresponding inhibitors may bring new opportunities for the treatment of tumors.
