# Supplementary material for: The crosstalk among the physical tumor microenvironment and the effects of glucose deprivation on tumors in the past decade
Source: Front Cell Dev Biol. 2023 Nov 1;11:1275543. doi: 10.3389/fcell.2023.1275543 (PMC10646288; doi:10.3389/fcell.2023.1275543)
Supplement: Supplementary file 1 [file Table1.pdf]

Supplementary Table S1 Studies of the effects of glucose deprivation on tumors and related important molecules and pathways in the past decade.

Cancer Type	Glucose Concentration	Key Gene	Full Name	Gene Function	Molecules	Pathways	Reference
Cancer	0mM	SLC7A11 (xCT)	Solute Carrier Family 7 Member 11	Death	F-actin	Disulfidptosis	(Liu et al., 2023)
Cancer	0mM	LncRNA HITT	HIF-1α inhibitor at the transcription level	Death	miR-106/HITT/PKM2/Lactate	Macrophage Polarization	(Zhao et al., 2022)
Cancer	0mM	SLC7A11 (xCT)	Solute Carrier Family 7 Member 11	Death	Inactivated AMPK	Metabolic homeostasis	(Lee et al., 2022)
Cancer	0mM	SIRT7	Sirtuin 7	Death	PCAF/MDM2/P53/P21	Cell cycle	(Lu et al., 2020)
Cancer	0mM	CHK1	Checkpoint Kinase 1	Death	AMPK/β-TrCP	Cell cycle	(Ma et al., 2019)
Cancer	0mM	ATF4	Activating Transcription Factor 4	Death	ERS/TRAIL	Apoptosis	(Iurlaro et al., 2017)
Cancer	0mM	AMPK	AMP-activated protein kinase	Survival	Arf6	Invasion/Migration	(Chen et al., 2022b)
Cancer	1mM	MDK	Midkine	Survival	LKB1-STRAD-Mo25/AMPK	Proliferation	(Xia et al., 2022)
Cancer	0mM	RSK	Ribosomal S6 kinase	Survival	CHK1	Cell cycle	(Ma et al., 2022)
Cancer	0mM	IDH1/ME1	Isocitrate Dehydrogenase (NADP(+)) 1 /Malic enzyme 1	Survival	NADPH generation	NADPH	(Ying et al., 2021)
Cancer	10mM	AKAP11	A-Kinase Anchoring Protein 11	Survival	cAMP/PKA/LC3	Autophagy	(Deng et al., 2021)
Cancer	0mM	MMP-9	Matrix Metallopeptidase-9	Survival	LKB1/AMPK/Keap1/Nrf2	Oxidative stress/Autophagy	(Endo et al., 2018)

Cancer	2.5mM	SRSF5	Serine And Arginine Rich Splicing Factor 5	Survival	HDAC1/CCAR1	Glycolysis	(Chen et al., 2018)
Cancer	2.5mM	LncRNA TRINGS	TP53-regulated inhibitor of necrosis under glucose starvation	Survival	P53/STRAP/GSK3β/NF-κB	Necrosis	(Khan et al., 2017)
Cancer	0mM	ME1	Malic Enzyme 1	Survival	NADPH/Pyruvate	NADPH	(Murai et al., 2017)
Cancer	1mM	PLD1	Phospholipase D1	Survival	FAO	ATP	(Cai et al., 2016)
Cancer	0mM	URI	Unconventional prefoldin RPB5 interactor	Survival	OGT/c-Myc	Proliferation	(Burén et al., 2016)
Cancer	1mM	PKA	cAMP-dependent protein kinase/Protein Kinase A	Survival	GSH	Glutaminolysis	(Palorini et al., 2016)
Cancer	0mM	c-Myc	MYC Proto-Oncogene	Survival	Serine synthesis pathway (SSP)/GSH	Metabolic homeostasis	(Sun et al., 2015)
Cancer	0mM	SAICAR	Succinylaminoimidazolecarboxamide ribose-5'-phosphate	Survival	PKM2	Glycolysis	(Keller et al., 2012)
Hepatoblastoma	0mM	LncRNA NBR2	LncRNA NBR2	Survival	miR-22/TCF7/GLUT1/HK2/PKM2/Snail/MMP-7	Glycolysis/EMT	(Zhu et al., 2021)
Hepatocellular carcinoma	5.5mM	HSF1	Heat Shock Transcription Factor 1	Survival	Snail1/E-cadherin	EMT	(Liu et al., 2016)
Hepatocellular carcinoma	2.5mM	IL-6	Interleukin 6	Survival	STAT3/GLUT1/GLUT3	Glycolysis	(Zhang et al., 2017a)
Hepatocellular Carcinoma	0mM	SESN2	Sestrin 2	Survival	Nrf2/ATF4/HK2	Glycolysis	(Li et al., 2023a)

Hepatocellular carcinoma	0mM	SND1	Staphylococcal Nuclease And Tudor Domain Containing 1	Survival	PGAM5	Mitophagy	(Liang et al., 2022)
Hepatocellular carcinoma	1mM	GDH1 (GLUD1)	Glutamate dehydrogenase 1	Survival	α-KG	Glutaminolysis	(Zhou et al., 2022)
Hepatocellular carcinoma	0mM	EZH2	Enhancer of zeste homolog 2	Survival	TRβ	Metabolic homeostasis	(Park and Lee, 2022)
Hepatocellular carcinoma	2.475mM	FUT1	Fucosyltransferase 1	Survival	ATF4/ACD147/ICAM-1/EGFR/EPHA2	Stemness	(Loong et al., 2021)
Hepatocellular carcinoma	0mM	Linc01564	Linc01564	Survival	miR-107/103a-3p/PHGDH	Glycolysis	(Zhang et al., 2021)
Hepatocellular Carcinoma	0mM	RHOF	Ras Homolog Family Member F, Filopodia Associated	Survival	AMPK/RAB3D/GLUT1/HK2/PDK1//LDH/EMT	Glycolysis/EMT	(Li et al., 2021a)
Hepatocellular carcinoma	0mM	SKP2	S-phase kinase-associated protein 2	Survival	P62/LC3/AMPK/CARM1	Autophagy	(Wei et al., 2018)
Hepatocellular carcinoma	0mM	Pinin	Pinin (PNN)	Survival	ERK1/2/PARP	Apoptosis	(Yang et al., 2016)
Hepatocellular carcinoma	0mM	PKA	cAMP-dependent protein kinase/Protein Kinase A	Survival	AMPK/P21	Cell cycle	(Ferretti et al., 2016)
Hepatocellular carcinoma	0mM	HRP-3	Hepatoma-derived growth factor (HDGF)-related protein-3	Survival	S6K1	Protein synthesis	(Cai et al., 2015)
Hepatocellular carcinoma	0mM	HBx	Hepatitis B virus (HBV) X protein	Survival	AMPK/ACC/FAO	ATP	(Wang et al., 2016a)
Hepatocellular carcinoma	0mM	PTG (PPP1R3C)	Protein Targeting To Glycogen	Survival	HO-1	Oxidative stress/Autophagy	(Yang et al., 2015)
Hepatocellular carcinoma	1mM	AhR	Aryl Hydrocarbon Receptor	Survival	ATF4/VEGF	Angiogenesis	(Terashima et al., 2013)

Hepatocellular Carcinoma	0mM/2.5mM	ARID1A	AT-rich interaction domain 1A	Death	USP9X/PRKAA2/ACC	Metabolic homeostasis	(Zhang et al., 2022b)
Hepatocellular carcinoma	5mM	PCK1 (PEPCK-C)	Phosphoenolpyruvate Carboxykinase 1	Death	AMPK/GFAT1/CHK2 O-GlcNAcylation	Cell cycle	(Xiang et al., 2021)
Hepatocellular carcinoma	5mM	SIRT1/SIRT6	Sirtuin1/Sirtuin6	Death	hnRNP A1/PKM2	Glycolysis	(Yang et al., 2019)
Hepatocellular carcinoma	5mM	TIP60 (KAT5)	Lysine Acetyltransferase 5	Death	P53/NOXA/PUMA	Apoptosis	(Fang et al., 2018)
Hepatocellular carcinoma	0mM	NDRG2	N-Myc Downstream Regulated Gene 2	Death	FAO	ATP	(Pan et al., 2017)
Hepatocellular carcinoma	0mM	NOX4	NADPH Oxidase 4	Death	ROS/AKT	Oxidative stress	(Owada et al., 2013)
Liver cancer	5mM	SIRT1	Sirtuin1	Survival	AMPK/P53/PGC1α	ATP	(Varghese et al., 2023)
Liver cancer	0mM	AEG-1	Astrocyte Elevated Gene-1	Survival	HIF-1α/VEGFC	Angiogenesis	(Umapathy et al., 2022)
Liver cancer	5mM	SESN2	Sestrin 2	Survival	PGC-1α	Glutaminolysis	(Kumar et al., 2018)
Liver cancer	0mM	CARM1 (PRMT4)	Coactivator Associated Arginine Methyltransferase 1	Death	GAPDH (R234 hypermethylation)	Glycolysis	(Zhong et al., 2018)
Pancreatic cancer	0.5mM	PKM2	Pyruvate Kinase M2	Survival	AMPKα1/BECN1/LC3	Autophagy	(Li et al., 2018)
Pancreatic cancer	0mM	ZFAS1	ZNFX1 antisense RNA 1	Survival	AMPK/ZEB1/E-cadherin/Vimentin	EMT	(Zhuo et al., 2023)
Pancreatic ductal adenocarcinoma	0mM	GPx1	Glutathione Peroxidase-1	Survival	ROS/AMPK/P62/LC3/GLUT1/HK2/PKM2/LDHA	Autophagy/Glycolysis	(Meng et al., 2018)

Pancreatic ductal adenocarcinoma	0mM	P53/TG2	P53/Transglutaminase2	Survival	ROS/BCL-2	Oxidative stress/Apoptosis	(Su et al., 2017)
Pancreatic ductal adenocarcinoma	0mM	CPT1A	Carnitine Palmitoyl Transferase 1A	Survival	NADPH/GSH/FAO	NADPH/ATP	(Luo et al., 2016)
Pancreatic ductal adenocarcinoma	2.5mM	IDH1	Isocitrate Dehydrogenase (NADP(+)) 1	Survival	NADPH	NADPH	(Vaziri-Gohar et al., 2022)
Breast Cancer	0mM	PGC-1α	Peroxisome proliferator-activated receptor-γ coactivator-1α	Survival	PMCA (Plasma membrane calcium ATPases)	Calcium homeostasis	(Park et al., 2023)
Breast cancer	0mM	PCK2 (PEPCK-M)	Phosphoenolpyruvate Carboxykinase 2	Survival	ATF4	Entosis	(Hyroššová et al., 2022)
Breast cancer	2.5mM	LncRNA HOXC-AS3	HOXC Cluster Antisense RNA 3	Survival	SIRT6	Metabolic homeostasis	(Zhu et al., 2022)
Breast cancer	2mM	HPIP	Hematopoietic PBX1-Interacting Protein	Survival	AMPK/SLC1A5 (ASCT2)/GLS	Glutaminolysis	(Penugurti et al., 2021)
Breast cancer	0mM	RASAL2	RAS Protein Activator Like 2	Survival	PP1MB/PRKAA(AMPKα)/PIK3C3/ATG14/BECN1	Autophagy	(Bao et al., 2021)
Breast cancer	0mM	GRP78	Glucose-Regulated Protein 78	Survival	UPR	ERS	(Xiao et al., 2019)
Breast cancer	0mM	Nrf2 (NFE2L2)	NFE2 Like BZIP Transcription Factor 2	Survival	P62	Oxidative stress/Autophagy	(Walker et al., 2018)
Breast cancer	0mM	KAP1(TRIM28)	KRAB domain-associated protein 1	Survival	MFN2	OXPHOS	(Cheng et al., 2016)
Breast cancer	0mM	PPARδ	Peroxisome Proliferator Activated Receptor Delta	Survival	PERK/CHOP	ERS/Oxidative stress	(Wang et al., 2016b)

Breast cancer	0mM	AKT	AKT Serine/Threonine Kinase	Survival	P53/LC3	Autophagy	(Sudhagar et al., 2016)
Breast cancer	0mM	NAMPT	Nicotinamide Phosphoribosyltransferase	Survival	NADPH	NADPH	(Hong et al., 2016)
Breast cancer	2.8 mM	ORP150	Oxygen-Regulated Protein of 150 kDa	Survival	β-galactosidase	Apoptosis/Senescence	(Kretowski et al., 2013)
Breast cancer	0mM	S6K1 (RPS6KB1)	Ribosomal Protein S6 Kinase B1	Survival	MCL-1/Survivin	Apoptosis	(Choi et al., 2013)
Breast cancer	0mM	LncRNA Vanguard	Vanguard	Survival	HMGB1	DNA repair	(Zhang et al., 2022a)
Breast cancer	0.5 mM	ZBP1	Z-DNA Binding Protein 1	Death	MLKL	Necroptosis	(Baik et al., 2021)
Breast cancer	0mM	SLC7A11 (xCT)	Solute Carrier Family 7 Member 11	Death	ROS	Oxidative stress	(Chen et al., 2020)
Breast cancer	0mM	PHB1	Prohibitin 1	Death	DRP1/BAX/BCL2/PARP	Apoptosis	(Raut et al., 2019)
Breast cancer	0mM	PHD2	HIF-prolyl-hydroxylase enzyme	Death	B55α	Apoptosis	(Di Conza et al., 2017b)
Breast cancer	0mM	NDRG2	N-Myc Downstream Regulated Gene 2	Death	AMPK/PARP	Apoptosis	(Kim et al., 2014)
Triple-negative breast cancer	5.5uM	miR-342-3p	miR-342-3p	Death	MCT1	Metabolic homeostasis	(Romero-Cordoba et al., 2018)
Lung adenocarcinoma	1mM	EZH2	Enhancer of zeste homolog 2	Survival	PDH3/HIF-1α/SLUG/SNAIL	EMT	(Saggese et al., 2023)

Lung adenocarcinoma	0mM	GFAT1	Glutamine-Fructose-6-phosphate Amidotransferase 1	Survival	TAB1/p38/P62/LC3	Autophagy	(Wei et al., 2022)
Lung adenocarcinoma	2.5mM	circZFR	Hsa_circ_0072088	Survival	MYO1B/AKT/MTOR	OXPHOS	(Ma et al., 2023)
Lung cancer	0mM	PFKP	Phosphofructokinase 1	Survival	AMPK/ACC2/FAO	ATP	(Chen et al., 2022a)
Lung cancer	0mM	KEAP1	Kelch Like ECH Associated Protein 1	Survival	Nrf2/SLC7A11(xCT)	Disulfidptosis	(Koppula et al., 2021)
Lung cancer	0mM	PGM1	Phosphoglucomutase 1	Survival	AMPK/HDAC8/ERK1/2	Metabolic homeostasis	(Li et al., 2020b)
Non-small cell lung cancer	0mM	4EBP1	4E Binding Protein 1	Death	PTPMT1/STAT3/BCL-2/MCL-1/Survivin	Apoptosis	(Wang et al., 2022)
Non-small cell lung cancer	0mM	LKB1 (STK11)	Liver Kinase B1	Survival	AMPK/ROS	Oxidative Stress	(Ren et al., 2021)
Non-small cell lung cancer	0mM	βIII-tubulin (TUBB3)	Tubulin Beta 3 Class III	Survival	GRP78/AKT/LC3	ERS/Autophagy	(Parker et al., 2016)
Non-small cell lung cancer	0mM	SESN2	Sestrin 2	Survival	ATF4/Nrf2/PARP	ERS/Apoptosis	(Ding et al., 2016)
Gastric cancer	5mM	HAP1	Huntingtin Associated Protein 1	Death	E-cadherin/N-cadherin/Vimentin/BCL-2/BAX/Caspase	EMT/Apoptosis	(Qu et al., 2023)
Gastric cancer	2.5mM	FDFT1	Farnesyltransferase 1	Death	miR-216a-5p/Lactate	Glycolysis	(Zhao et al., 2021)
Gastric cancer	0mM	DLC3 (STARD8)	Deleted In Liver Cancer 3 Protein(StAR Related Lipid Transfer Domain Containing 8)	Death	RhoA/JNK/AP-1/MACC1/Glucose chemotaxis	Glycolysis	(Lin et al., 2019)

Gastric cancer	0mM	MACC1	MET Transcriptional Regulator	Survival	HK/PDK/LDH	Glycolysis	(Lin et al., 2015)
Gastric cancer	2.5mM	UCH-L3	Ubiquitin C-terminal hydrolase-L3	Survival	c-Fos/ β-catenin	Stemness	(Lee et al., 2023)
Gastric cancer	0mM/2.5mM	SIRT1	Sirtuin1	Survival	FoxO1/Rab7/LC3/P62/BECN1	Autophagy	(Zhu et al., 2023)
Colon cancer	1mM	ACSS2	Acyl-CoA Synthetase Short Chain Family Member 2	Survival	Cbp/HIF-2α/VEGFA/MMP9	Invasion/Migration	(Garcia et al., 2023)
Colon cancer	0mM	NRP1	Neuropilin1	Survival	AKR1B10/GAPDH/LC3	Autophagy	(Li et al., 2021b)
Colon cancer	0mM	JMJD2B	Jumonji domain-containing protein 2B	Survival	ERK/GLUT1	Glycolysis	(Fu et al., 2018)
Colon cancer	0mM	HIF-1α	Hypoxia-inducible factor-1α	Survival	STAT3/TCF4/PARP	Apoptosis	(Nishimoto et al., 2014)
Colorectal cancer	0mM	EZH2	Enhancer of zeste homolog 2	Death	GLS/GSH	Glutaminolysis	(Liu et al., 2021)
Colorectal cancer	0mM	TRAIL	Tumor necrosis factor-Related Apoptosis-Inducing Ligand	Death	ATF4/CHOP/PUMA/DR5	ERS/Apoptosis	(Kalimuthu et al., 2021)
Colorectal cancer	0mM/1mM	LncRNA NBR2	Neighbor Of BRCA1 LncRNA 2	Death	AMPK/mTOR	Metabolic homeostasis	(Yu et al., 2019)
Colorectal cancer	0mM	HMGB1	High Mobility Group Box1	Death	RAGE/TLR4/PI3K/MEK	Invasion/Migration	(Sharma et al., 2016)
Colorectal cancer	0mM	SIRT1	Sirtuin1	Survival	FAO	ATP	(Wei et al., 2023)
Colorectal cancer	0mM	JMJD2B	Jumonji domain-containing protein 2B	Survival	LC3	Autophagy	(Tan et al., 2020)

Colorectal cancer	2.5mM	RPIA	Ribose 5-Phosphate Isomerase A	Survival	CARM1/Pentose phosphate pathway (PPP)/NADPH	NADPH	(Guo et al., 2020)
Colorectal cancer	0mM	LncRNA GLCC1	GLCC1	Survival	HSP90/c-Myc/LDHA	Glycolysis	(Tang et al., 2019)
Colorectal cancer	0mM	Pim1	Pim1 (PIM1)	Survival	HK2/LDHA	Glycolysis	(Zhang et al., 2018)
Colorectal cancer	0.5mM/1.5mM	ATF4	Activating Transcription Factor 4	Survival	MDR1	Drug resistance	(Hu et al., 2016)
Colorectal cancer	0mM	Trx-1	Thioredoxin 1	Survival	G6PD/E-cadherin/Vinmentin	NADPH/EMT	(Lu et al., 2022)
Glioblastoma	5mM	SHC3	SHC adaptor protein 3	Survival	GLUT	Glycolysis	(Azzalin et al., 2020)
Glioblastoma	0mM	EphA2	EPH Receptor A2	Survival	SLC7A11 (xCT)/ROS/ERK/RSK	Proliferation	(Teramoto and Katoh, 2019)
Glioblastoma	0mM	ID2	Inhibitor of DNA binding 2	Survival	ROS	Oxidative stress	(Zhang et al., 2017b)
Glioblastoma	0mM	SLC7A11 (xCT)	Solute Carrier Family 7 Member 11	Death	ROS	Oxidative stress	(Yamamoto et al., 2021)
Glioblastoma	0mM	SLC7A11 (xCT)	Solute Carrier Family 7 Member 11	Death	ROS	Oxidative stress	(Yamaguchi et al., 2020)
Glioblastoma	0mM	IL-11Rα	Interleukin 11 Receptor Subunit Alpha	Survival	GLUD1/GSS/c-Myc	Glutaminolysis	(Stuart et al., 2023)
Glioma	0mM/0.1mM	AMPK	AMP-activated Protein Kinase	Survival	ATP	Metabolic homeostasis	(Belo do Nascimento et al., 2022)

Glioma	0mM	B55γ (PPP2R2C)	Protein Phosphatase 2 Regulatory Subunit Bgamma	Survival	SIK2/S6K	Protein synthesis	(Li et al., 2015)
Glioma	0mM	SIRT6	Sirtuin6	Death	HK2/Nrf2/XOR	Oxidative stress	(Sheikh et al., 2018)
Glioma	2mM/5mM	FOXO3a	Forkhead Box O3	Death	TRAIL	Apoptosis	(Brucker et al., 2016)
Renal Cancer	0mM	AMPK	AMP-activated Protein Kinase	Death	JAK2/STAT3/P53/GPX4	Ferroptosis	(Li et al., 2022)
Renal cancer	0mM	UAP1	UDP-N-acetylglucosamine Pyrophosphorylase 1	Death	P53/P21/Bip/XBP1	Cell cycle/ERS	(Isono et al., 2014)
Renal cell carcinoma	2.5 mM	AMPK	AMP-activated Protein Kinase	Survival	PKM2/CCND1/c-Myc	Proliferation	(Liu et al., 2019)
Ovarian cancer	0mM	LSR	Lipolysis Stimulated Lipoprotein Receptor	Survival	LKB1/AMPK/PARP	Apoptosis	(Takahashi et al., 2021)
Ovarian cancer	0.69 mM	NNMT	Nicotinamide N-Methyltransferase	Survival	ZEB1	Metabolic homeostasis	(Kanska et al., 2017)
Gallbladder carcinoma	2.5mM	Stathmin1 (STMN1)	Stathmin1	Survival	hKIS/p27/E2F1	Invasion/Migration	(Wang et al., 2021)
Gallbladder carcinoma	0.75 Mm	MOB1A	MOB kinase activator 1A	Survival	IL6/STAT3/P62/BECN1/LC3/PARP	Autophagy/Apoptosis	(Yang et al., 2020)
Papillary thyroid carcinoma	0mM	CPT1C	Carnitine Palmitoyltransferase 1C	Survival	AMPK	ATP	(Wang et al., 2017)
Thyroid Carcinoma	0mM	SKP2	S-phase kinase-associated protein 2	Survival	PHLPP1/AKT/P62/LC3	Autophagy	(Shao et al., 2023)
Cholangiocarcinoma	0mM	LGR5	Leucine Rich Repeat Containing G Protein-Coupled Receptor 5	Survival	AKT/ROS	Stemness	(Yoshikawa et al., 2019)

Esophageal squamous cell carcinoma	0mM	ASNS	Asparagine Synthetase (Glutamine-Hydrolyzing)	Survival	Nrf2/ATF4	Metastasis	(Fang et al., 2020)
Neuroblastoma	0mM	TRAP-1	Tumor Necrosis Factor Receptor-associated Protein 1	Survival	HIF-2α/SLC1A5/GLS	Glutaminolysis	(Dharaskar and Amere Subbarao, 2023)
Osteosarcoma	1mM	LncRNA HAND2-AS1	HAND2 Antisense RNA 1	Death	FBP1/HIF-1α/GLUT1/HK2/ALDOC/MCT4	Glycolysis	(Kang et al., 2018)
Cervical cancer	0mM	MAT2A	Methionine Adenosyltransferase 2A	Survival	AMPK/PDCD6 methylation/BCL-2/BAX/Caspase	Apoptosis	(Luo et al., 2022)
Prostate cancer	0mM	GLUT1 (SLC2A1)	Solute Carrier Family 2 Member 1	Survival	ROS/SOD2/GSH	Oxidative stress	(Gonzalez-Menendez et al., 2018)
